# Supplementary material for: Small Gold Nanorods with Tunable Absorption for Photothermal Microscopy in Cells
Source: Adv Sci (Weinh). 2016 Nov 19;4(2):1600280. doi: 10.1002/advs.201600280 (PMC5323823; doi:10.1002/advs.201600280)
Supplement: Supplementary file 1 — Supplementary [file ADVS-4-na-s001.pdf]

Copyright WILEY-VCH Verlag GmbH & Co. KGaA, 69469 Weinheim, Germany, 2016.

Supporting Information

for *Adv. Sci.,* DOI: 10.1002/advs.201600280

Small Gold Nanorods with Tunable Absorption for Photothermal Microscopy in Cells

*Edakkattuparambil Sidharth Shibu, Nadezda Varkentina, Laurent Cognet, and Brahim Lounis**

Supporting Information

Gold nanorods with tunable absorption for photothermal microscopy in cells

*Edakkattuparambil Sidharth Shibu[1,2], Nadezda Varkentina[1,2], Laurent Cognet[1,2] and Brahim Lounis[1,2,]**

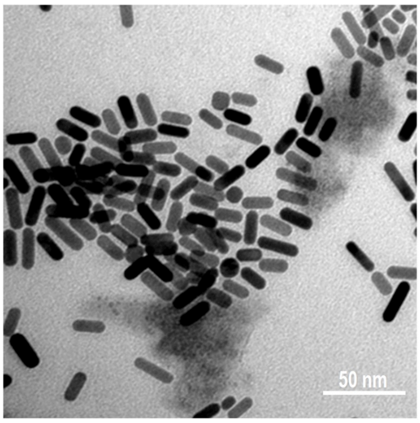

Figure S1. TEM image of nanorods collected from a fraction of rods having a larger aspect ratio than in Figure 2.

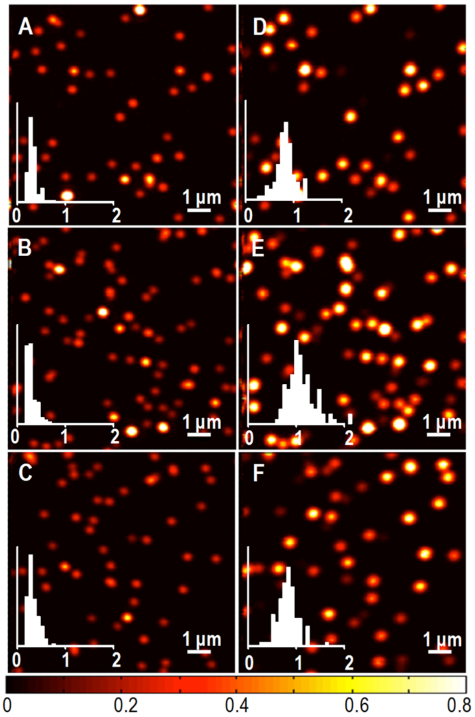

Figure S2. PhI images of nanorods from fractions with different aspect ratio (peak SPR at 634 nm, 648 nm and 655 nm respectively) excited with beams at 532 nm (A-C) and 640 nm

(D-F). Insets: Corresponding PhI signal histograms constructed from more than 100 single nanorods.

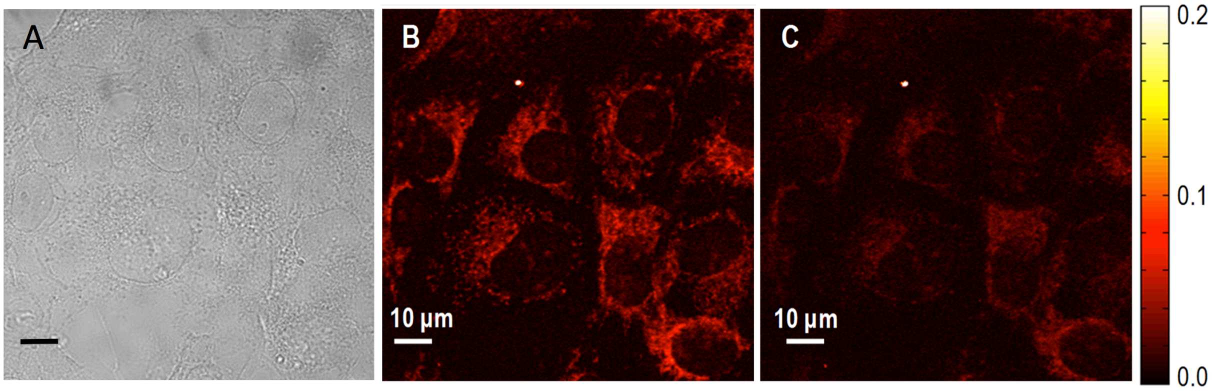

Figure S3. (A) White light and (B and C) PhI of COS 7 cells under (B) 532 nm and (C) 640 nm excitations. PhI under 532 nm excitation shows larger mitochondrial absorption compared to 640 nm excitation.

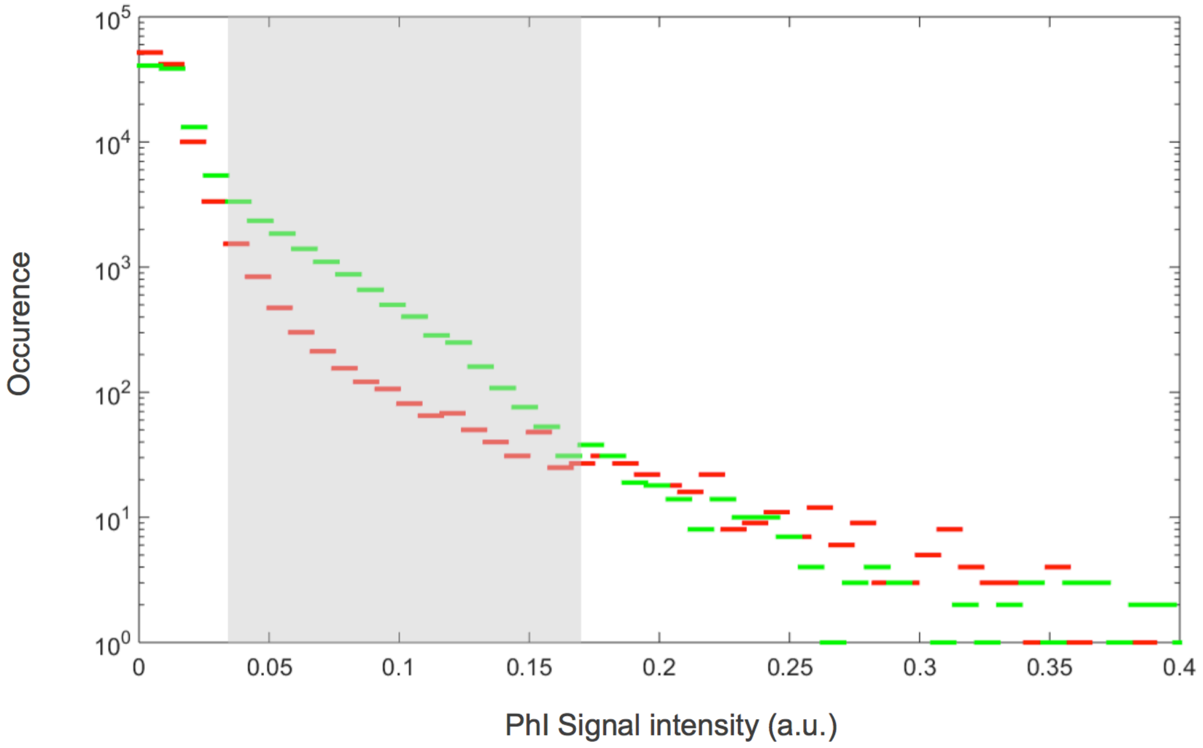

Figure S4. Histograms of PhI signals of images displayed in Figure 4: Green COS 7 cells are excited with 532 nm laser, Red excitation at 640 nm. Clearly, PhI under 532 nm excitation shows larger mitochondrial signals (gray area) compared to 640 nm excitation.
